# Toward an Ethical Framework for the Text Mining of Social Media for Health Research: A Systematic Review

**DOI:** 10.3389/fdgth.2020.592237

**Published:** 2021-01-26

**Authors:** Elizabeth Ford, Scarlett Shepherd, Kerina Jones, Lamiece Hassan

**Affiliations:** ^1^Department of Primary Care and Public Health, Brighton and Sussex Medical School, Brighton, United Kingdom; ^2^Population Data Science, Medical School, Swansea University, Swansea, United Kingdom; ^3^Division of Informatics, Imaging & Data Sciences, University of Manchester, Manchester, United Kingdom

**Keywords:** social media, text-mining, health research, natural language processing, ethics

## Abstract

**Background:** Text-mining techniques are advancing all the time and vast corpora of social media text can be analyzed for users' views and experiences related to their health. There is great promise for new insights into health issues such as drug side effects and spread of disease, as well as patient experiences of health conditions and health care. However, this emerging field lacks ethical consensus and guidance. We aimed to bring together a comprehensive body of opinion, views, and recommendations in this area so that academic researchers new to the field can understand relevant ethical issues.

**Methods:** After registration of a protocol in PROSPERO, three parallel systematic searches were conducted, to identify academic articles comprising commentaries, opinion, and recommendations on ethical practice in social media text mining for health research and gray literature guidelines and recommendations. These were integrated with social media users' views from qualitative studies. Papers and reports that met the inclusion criteria were analyzed thematically to identify key themes, and an overarching set of themes was deduced.

**Results:** A total of 47 reports and articles were reviewed, and eight themes were identified. Commentators suggested that publicly posted social media data could be used without consent and formal research ethics approval, provided that the anonymity of users is ensured, although we note that privacy settings are difficult for users to navigate on some sites. Even without the need for formal approvals, we note ethical issues: to actively identify and minimize possible harms, to conduct research for public benefit rather than private gain, to ensure transparency and quality of data access and analysis methods, and to abide by the law and terms and conditions of social media sites.

**Conclusion:** Although social media text mining can often legally and reasonably proceed without formal ethics approvals, we recommend improving ethical standards in health-related research by increasing transparency of the purpose of research, data access, and analysis methods; consultation with social media users and target groups to identify and mitigate against potential harms that could arise; and ensuring the anonymity of social media users.

## Introduction

In the last two decades, social media platforms, social networking sites, and internet discussion forums have undergone an exponential increase in users, with 3.48 billion active users in 2019, a 9% increase from the previous year (1). Facebook continues to be the highest-ranking social media website, with 2.27 billion active users per month, while Twitter attracts around 326 million active users per month (1). Other sites such as Instagram (nearly 1 billion users) and TikTok are rapidly gaining users, particularly of a younger demographic. Additionally, many discussion forums on the internet provide ways for users to discuss and share their experiences and seek advice from their peers.

Social media platforms are used to disseminate health information (2, 3) and are used by health care professionals to interact with and advise users (4, 5). In addition, users post information about their health behaviors, experiences, and attitudes, often in publicly open forums (4, 6). With 57% of the world's population accessing social media each year (1), this opens new opportunities for researchers to harvest and analyze data for health research, gaining information regarding people's health on a scale that would have previously been unachievable (6). This new source of health data may also give researchers access to the views and experiences of people who have traditionally been hard to recruit to research studies (7). The availability of this information is already being utilized by health researchers, to study adverse drug effects for pharmacovigilance (8, 9), flu outbreak surveillance (10), and mental health monitoring (11).

The vast amount of data available from social media and patient discussion forums and the necessity of identifying relevant posts from a large number of irrelevant ones mean that computer-based text-mining techniques are often used. Natural language processing (NLP) is a branch of computer science that uses series of rules and/or machine learning algorithms to identify relevant information from written natural language. Usually, algorithms are able to recognize named entities to a high accuracy and also assess the context for negation, subject, timing, and hedging (12). Further work, such as looking for links between drugs and side effects, focuses on extracting relationships between entities within the same sentence or document (13). Usually, quantitative data are derived from this information extraction, which can then be analyzed statistically, allowing simultaneous analysis of multiple posts or documents. Some research using social media data has been qualitative and thematic, which involves more detailed and in-depth reading and analysis of the full written content (14).

NLP research uses the available text, which is posted on discussion forums and social media; as it usually does not involve any interaction or direct contact with social media users, it is assumed to be ethically low risk. It is also perceived as a “low-stakes” approach for student projects, and therefore, the research community may include many new or inexperienced researchers who are not well-versed in ethical issues. While medical researchers are usually trained in “human subjects” research ethics, computer science researchers may be less experienced with key ethical issues (15). In addition, previous controversial incidents have raised important questions about the ethics and acceptability of this approach. In 2008, researchers published data that they had collected from the Facebook accounts of an entire cohort of university students over 4 years. This research subsequently came under intense scrutiny when it was discovered that the “anonymous” university used in the study could be easily re-identified (16). In a second controversial case in 2016, Danish researchers published data from 70,000 OkCupid users, with information including usernames, age, gender, location, and sexual preference being made publicly available with no attempts to anonymize the data (17).

Such incidents have led to concern that individuals' privacy could be threatened, with only a small amount of effort from an adversary, if harvested datasets are made public. Previous studies reviewing public and patients' views have indicated the fears of harms that patients have when their sensitive, personal health data are used for the secondary purpose of research (18, 19). Participants feared that if they were re-identified from their data following unauthorized disclosure or access, this could lead to identity theft, consequences for employment, pension eligibility, increased insurance costs, social discomfort, community embarrassment, unnecessary stigmatizing judgments in clinical settings, or the use of their data for financial gain (19). Although these studies focused on clinical and administrative data sources, it is possible that social media users may have similar fears about potential consequences of the secondary use of their internet posts.

However, personal content posted on social media platforms and internet discussion forums has been made public to a greater or lesser extent by the content creator and therefore differs considerably from clinical data created by a health care professional in the course of recording a confidential consultation. It is therefore important to have a separate framework guiding best practice for using this type of health data in research. Core research ethics principles for biomedical research, such as those proposed in the Belmont Report (20), and Beauchamp and Childress (21), and for ICT research, such as proposed in the Menlo Report (22) are likely to be relevant, as they give overarching principles that are relevant for many scenarios in health research. Beauchamp and Childress' four main principles have been shown to underpin public or lay thinking about ethical issues in data-sharing for health research (19).

Previously, researchers have acknowledged the lack of ethical guidance in social media data mining (6, 23) and often institutional ethics review boards report feeling ill-equipped to keep pace with rapidly changing technologies used in research (24, 25). We therefore aimed to review the literature on best ethical practice in this field to bring together recommendations for text-mining/NLP researchers who are using social media and patient discussion forum data for health research. We cast our net wide for this study, systematically searching both academic and gray literature, and aiming to include social media users' perspectives in our recommendations.

## Materials and Methods

A systematic review protocol was registered with the PROSPERO database (CRD42018112923) (26). Three systematic searches were undertaken in parallel, two are reported using PRISMA flow charts (27), and the manuscript reporting adheres to the Enhancing Transparency in Reporting the Synthesis of Qualitative Research (ENTREQ) statement (28). No ethical approval was sought for this study as it involved analysis of previously published reports.

### Search Strategy

We conducted three systematic searches, two of academic literature and one of the gray literature.

#### Search 1 and 2: Academic Literature (1: Commentaries, Editorials, Tutorials, and Recommendations; 2: Qualitative Studies Reporting Social Media Users' Views on Social Media Text Mining for Health Research)

Three databases were searched: MEDLINE (Ovid), Scopus, and ASSIA (Proquest). The terms used in the search are given in Table 1. Truncations and Boolean operators were used to allow for a comprehensive but specific search. The search was limited to results published in English, with no constraint on the country of publication. A date restriction of 2006–2018 was employed, as this was when Facebook was expanded beyond educational institutions and made public to anyone with a registered email address, changing the landscape of online social networking. Results from each search were imported into separate Zotero files and duplicates were discarded. Reference screening was conducted to identify further papers.

**Table 1:** Search date, search strings, and databases for three searches.

	**Commentaries, Editorials, Tutorials, and Recommendations**	**Qualitative studies on Social Media Users' Views**	**Gray Literature**
Search Date	18.10.2018	30.10.2018	18.08.2019
Search String	(moral OR ethic*) AND (“social medi*” OR “discussion forum*” OR twitter OR “social listening” OR “social networking” OR facebook) AND (health* OR medical) AND (research OR data OR evaluation* OR experiment*)	(patient* OR public OR community) AND (attitude* OR opinion* OR thought* OR idea* OR feeling* OR mindset* OR view* OR position* OR understanding* OR perspective* OR belief*) AND (“social medi*” OR twitter OR “discussion forum*” OR facebook OR “social networking” OR “social listening”) AND (health* OR medical) AND (research OR data OR evaluation* OR experiment*)	(ethic* AND (“social media” OR “patient forum” AND “discussion forum”) AND “health research”) “social media research ethics”; “social media ethics guidelines”; “guidelines on social media mining”; “ethical issues in social media research”; “social media mining for health research ethics”
Databases	MEDLINE (Ovid), Scopus and ASSIA (Proquest)	MEDLINE (Ovid), Scopus and ASSIA (Proquest)	Google

#### Search 3: Gray Literature

What is meant by gray literature is undefined, but is generally recognized to be publications and documents not controlled by commercial organizations or publishers, or not collected and preserved in library holdings (29). We searched for these documents using the Google search engine; search terms are given in Table 1. The first 60 results were examined.

### Inclusion and Exclusion Criteria

Due to the qualitative nature of this research and its exploration of a phenomenon rather than an intervention, the SPIDER tool (30) was used to construct the inclusion and exclusion criteria for academic articles (Table 2). Following each search, one author (SS) screened each set of articles based on their title and abstract. The remaining articles from each search were then screened using the full text by two authors (SS and EF), with a consensus being met for each article regarding its eligibility.

**Table 2:** Inclusion and exclusion criteria for systematic review using the SPIDER tool.

	**Inclusion criteria**	**Exclusion criteria**
**Commentaries, editorials, tutorials, and recommendations**		
Sample	No human sample necessary	Empirical studies recruiting and gathering data directly from human participants
Phenomenon of Interest	Ethical considerations of re-using social media post containing health information for health research. Can be primary or secondary focus	Focusing on non-social media technology such as search engines and wearable technology Focus on interventions using social media Focus on delivery of health care advice via social media
Design	Any expert opinion papers including: commentaries, opinion, perspective, discussion, editorial, literature review papers etc.	Empirical/experimental methods
Evaluation	Any qualitative exploration of the ethical considerations toward using social media posts for health research, in any part of the paper	Quantitative evaluation of results
Research Type	Peer-reviewed journal articles in English published from 2006 to 2018	Quantitative research Not in peer-reviewed journal Published in a language other than English Conference Abstracts
**Qualitative studies on social media users' views**		
Sample	People who post health information on social media. No minimum sample size required	Focused on other populations' views, such as researchers or review board members
Phenomenon of Interest	Views or attitudes on the use of health information posted on social media being utilized in health research	Not specific to health research Focus on interventions using social media
Design	Qualitative or mixed methods studies including surveys, questionnaires, interviews, and focus groups	Studies with only quantitative data.
Evaluation	Qualitative analysis of views or attitudes toward the use of health-related social media posts being re-used for health research	Quantitative evaluation only
Research Type	Peer-reviewed journal articles in English with qualitative or mixed methods, published from 2006 to 2018	Systematic reviews, editorials, commentaries, opinion, perspective, discussion papers, etc. Not published in a peer-reviewed journal Published in a language other than English Conference Abstracts
**Gray literature**		
Literature type	Reports giving commentaries, editorials, tutorials, guidelines, or recommendations	Digital tools, websites, e.g., showcasing research groups, social media sites themselves. PowerPoint slides
Topic	Ethical health or social science research, which uses social media data in a passive way (e.g., data scraping)	Recruitment or interaction with social media users; interventions or health care delivered or deliverable through social media Guidance of use of ethical social media by health care professional Research for commercial companies, e.g., social listening, market research. NHS patient data

For the gray literature search, articles were accepted if they were published commentaries, editorials, tutorials, guidelines, or recommendations for ethical health or social science research, which used social media data in a passive way (e.g., qualitative analysis of posts; text mining), i.e., not involving recruitment or interaction with social media users, or providing any interventions through social media. Screening was conducted by EF and reports were checked for eligibility by LH. Where this search brought up academic publications, these were examined according to search 2 criteria and, if eligible, were added to the pool of academic articles covering commentaries, editorials, tutorials, and recommendations.

### Quality Assessment

Academic articles reporting qualitative studies on social media users' views were assessed for quality by author SS using the Mixed Methods Appraisal Tool (MMAT) (31). For a study to be included in the final review, it had to score at least three points, with two points coming from the initial two screening questions. All eligible studies met this criterion.

### Data Extraction and Synthesis

The following information was extracted from qualitative studies (where relevant): date, location, and publication type, authors, data, study design, number of participants, research objective, and findings.

For thematic analysis, all articles and documents were imported into NVivo 12, and separate thematic analyses were carried out for commentaries, editorials, tutorials, and recommendations and social media user studies, following the thematic synthesis principles of Thomas and Harden (32). These principles allow for transparency and reproducibility of the methods due to its detailed methodology. Analysis involved coding all relevant text, line by line into nodes (initial coding was conducted by SS). Existing nodes were used for subsequent papers where appropriate, and new nodes were created where necessary. Once a full coding and node structure had been completed, nodes were examined and discussed between SS and EF. Nodes were then aggregated into larger descriptive themes, and following iterative refinement and discussion, these were then used to deductively generate the final analytical themes for the results of the study. When synthesizing the findings, priority was given to data that contributed to the formation of a set of ethical guidelines. Once the two types of academic articles had each been analyzed separately, the themes generated for each set were examined and matched together. Next, the gray literature results were examined in the same way, identifying extracts that related to existing themes from the previous two analyses and coding extracts that related to new themes (coding was conducted by EF, and nodes were examined and discussed by LH). Once all gray literature documents had been coded, a final complete set of themes was agreed by all authors. All articles and documents were then re-read to ensure all content relating to the final themes was extracted.

## Results

### Search Results

#### Search 1: Academic Articles Comprising Commentaries, Editorials, Tutorials, and Recommendations

**Figure 1:**
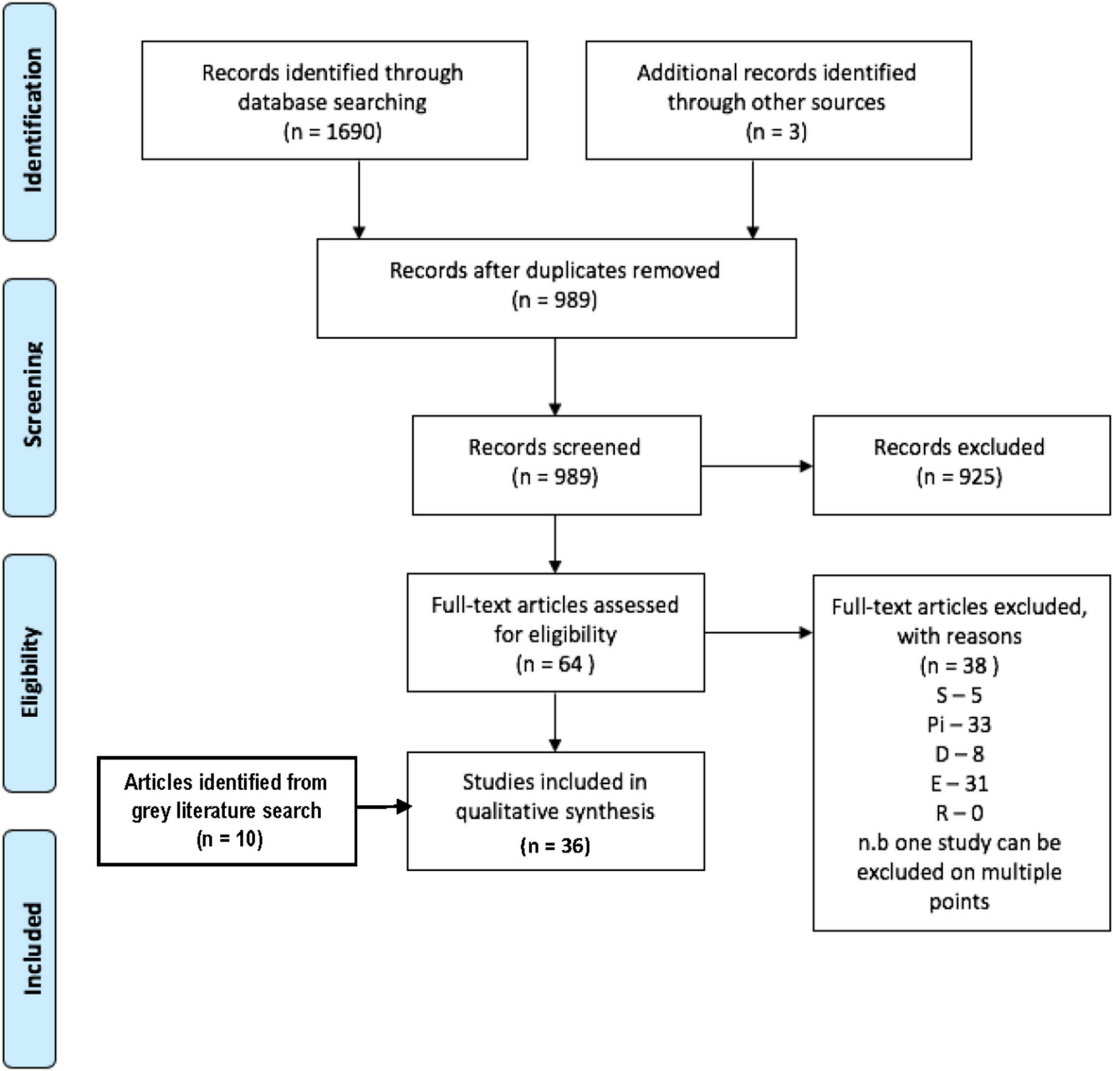
Prisma flow chart for inclusion of articles for Search 1.

From 1,690 articles returned by the search and by reference screening, 26 met eligibility criteria and were included in the study. A further 9 articles and a book chapter were identified as eligible from the gray literature search, making 36 articles in total (Figure 1). These were perspective, commentary, or recommendation full articles (*N* = 20), literature/systematic reviews (*N* = 7), case studies (*N* = 5), opinion (*N* = 1), conference proceedings (*N* = 1), book chapter (*N* = 1), and editorial (*N* = 1) papers. All papers were published between 2008 and 2019, with 31 of the articles published from 2013. Authors of the papers were geographically spread between the USA (*N* = 15), UK (*N* = 13), Germany (*N* = 2), Switzerland (*N* = 2), Canada (*N* = 1), France (*N* = 1), Australia (*N* = 1), and Saudi Arabia (*N* = 1).

#### Search 2: Qualitative Studies of Social Media Users' Views

**Figure 2:**
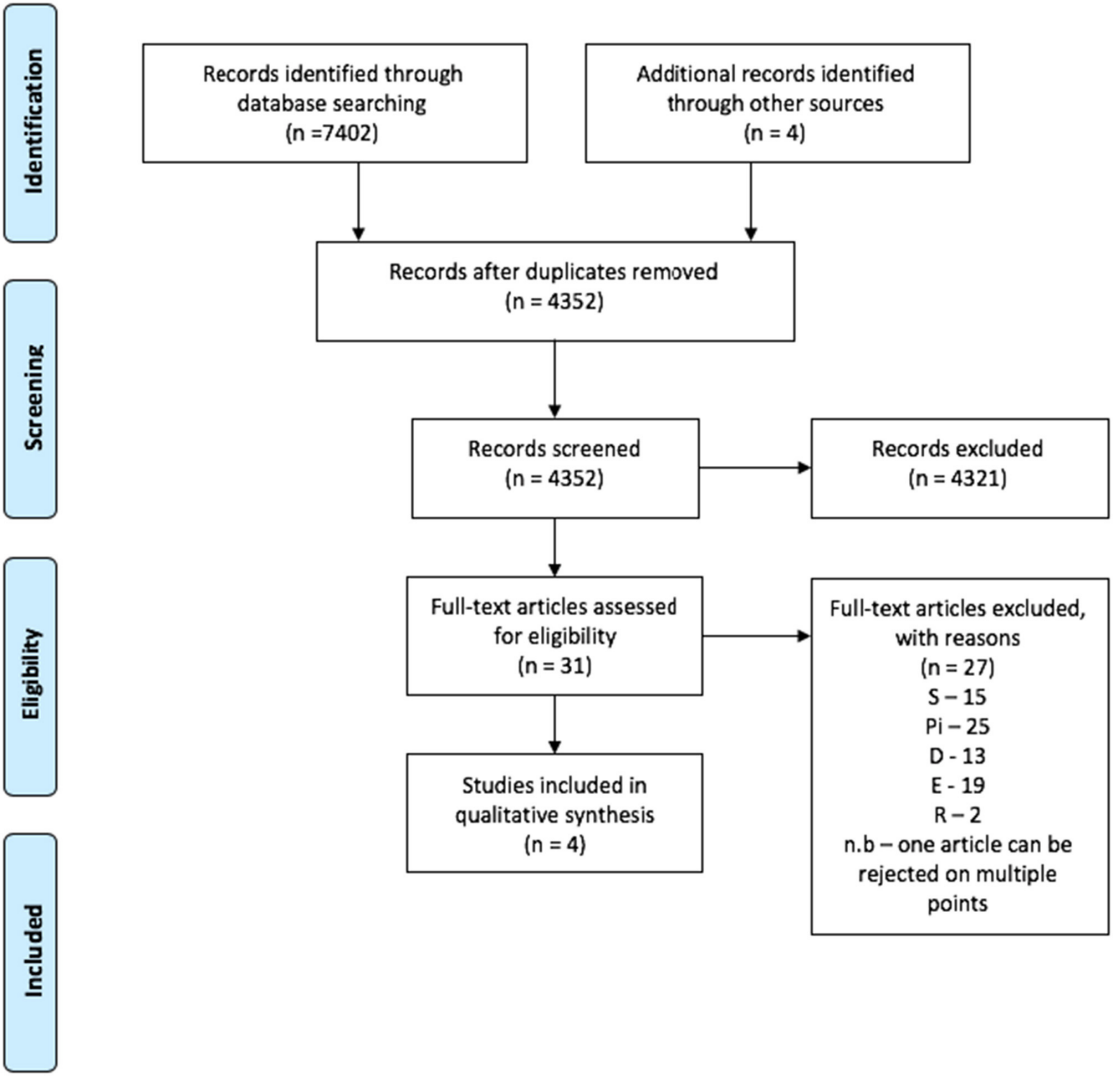
Prisma flow chart for inclusion of articles for Search 1.

A total of 7402 peer reviewed articles were identified through the systematic search, with an additional 4 articles identified though reference screening. Of these, four papers met the inclusion criteria and were included in the review (Figure 2). All studies were published after 2012 and were geographically spread between the USA (*N* = 2), the UK (*N* = 1), and Australia (*N* = 1). All studies were qualitative and ranged in the number of participants from 26 to 132, with a total of 232 participants across all studies. All studies published some demographic information, with males and females being well-represented. Three of the four studies included the average age or range of ages for participants, with two of the studies focusing on adolescent populations. Two of the studies focused on specific conditions, one being diabetes and the other mental health. Study characteristics are shown in Table 3.

**Table 3:** Study characteristics for qualitative studies of social media users.

**References**	**Location**	**Study design**	**Number of participants**	**Demographics of participants**	**Research objective**	**Findings**
Bond et al. (33)	UK	Qualitative online semi-structured asynchronous interviews	26	46% male 35% female 19% unidentified	To assess the opinions of diabetes discussion board users concerning their views about health researchers using their posts	Aggregated data can be used by researchers, but no consensus on the views of using individual information
Mikal et al. (34)	USA	Semi-structured focus group interviews	26	65% male 31% female 4% unidentified Average age, 26.9 years	To assess public views toward using Twitter data for population mental health monitoring	Relatively positive view to using Twitter data provided data are aggregated
Monks et al. (35)	Australia	Focus group interviews	48	Aged 13–14	To assess how young people perceive the use of social media for health and well-being research	Concerns regarding privacy, consent, and practicality but also recognize the benefits, and are open to social media research if appropriate consent and confidentiality were ensured
Moreno et al. (36)	USA	Interviews following public Facebook profiles being identified	132	48.8% male 51.2% female Average age, 18.4 years	To assess how older adolescents feel regarding being identified for a study via there Facebook profiles	Most adolescents had a positive view toward the use of Facebook for research, but those who were uneasy or concerned showed confusion toward profile security settings

#### Search 3: Gray Literature

The separate search strings produced a range of different and overlapping results, so the full number of results screened is not available. After identifying and examining 12 reports in detail, 7 were identified as eligible. From the gray literature search, a further nine academic articles and one book chapter, not picked up by search 1, were also found to be eligible and added to the pool of articles from search 1. The seven included gray reports were dated between 2012 and 2016; five were authored by researchers in the UK and two had international authorship.

### Themes

A total of 47 articles were therefore included for thematic analysis, from the three sources: 36 commentaries, 4 qualitative studies, and 7 gray literature reports. During the thematic analysis, four themes were identified from the qualitative studies of user views: private vs. public data; consent; anonymity; and research for public benefit. Six themes were separately identified from the academic articles comprising commentaries, editorials, tutorials, and recommendations; these were private vs. public data; consent; anonymity; weighing harms against benefits, necessity for ethical approval for studies; and governance of data, annotations, algorithms, and linkage. Three of these themes overlapped, giving seven from academic literature. These themes were largely supported by the gray literature, and an eighth theme was identified from these reports: legal issues and terms and conditions of social media sites. The reports and articles contributing to each theme are shown in Table 4.

**Table 4:** Contribution of each article or report to the eight themes.

**Article**	**Are data private or public?**	**Consent—should users be asked?**	**Anonymity**	**Weighing harms against benefits**	**Research for public benefit**	**Legal concerns and site T&Cs**	**Governance of data, annotations, algorithms, and linkage**	**When is ethical approval needed?**
**Gray literature reports, recommendations, and guidelines**								
AoIR recommendations (37)	X			X				X
ESOMAR guideline on Social Medial Research (38)		X	X	X	X	X		
Government Social Media Research Group (39)	X	X	X	X	X			
Natcen users' views (40)		X	X	X	X			
Sheffield workshop (41)	X	X	X	X		X		
Townsend and Wallace Aberdeen (42)	X	X	X	X		X		
Wisdom of the crowd (43)		X						
**Academic literature comprising commentaries, editorials, tutorials, and recommendations**								
Ahmed et al. (book chapter) (44)	X	X		X		X		X
Azam (45)	X	X						
Azer (46)		X		X				
Benton et al. (47)	X		X				X	X
Bica and Anderson (48)	X	X	X	X		X		
Boyd and Crawford (49)		X						X
Chiauzzi and Wicks (50)		X	X			X	X	X
Conway (51)		X	X	X				
Conway and O'Connor (52)		X		X				
Conway et al. (12)			X		X			X
Denecke (53)			X					
Farnan (54)				X				
Goodyear (55)								X
Gustafson and Woodworth (56)	X	X	X	X				
Hammer (57)		X						
Hunter et al. (58)	X	X	X	X				
Lafferty and Manca (59)		X	X					X
Li (60)		X		X				
McKee (61)		X	X				X	
Moreno et al. (62)	X							
Moreno et al. (63)	X		X					X
Norval and Henderson (64)	X							
Ravoire et al. (65)			X					
Schneble et al. (66)	X							
Sharkey et al. (67)	X		X					
Smith and Milnes (68)			X					
Spriggs (69)	X	X	X					
Sugiura et al. (70)	X	X	X					
Swirsky et al. (71)	X							
Taylor and Pagliari (72)	X							
Taylor et al. (73)								X
Valente and Pitts (74)			X	X				
Vayena et al. (75)	X							
Weigmann (76)								X
Williams et al. (77)	X		X			X	X	
Wongkoblap et al. (78)								X
**Studies of social media users' views**								
Bond et al. (33)		X	X		X			
Mikal et al. (34)	X	X	X		X			
Monks et al. (35)	X	X	X		X			
Moreno et al. (36)	X	X			X			

#### Private vs. Public Data—To Whom Does the Data Belong?

One of the leading themes to emerge from the included articles was the argument around whether social media posts should be considered public or private data. Many commentators argued that once data have been openly posted on social media, it then becomes part of the public domain, and that subsequently dismantles the expectation of privacy and implies consent for the use of data for any purpose (45, 51, 52, 56, 57, 61, 69).

“*However, those posting information do so knowing it is an open public forum; therefore, researchers may conclude that consent is implied for the use of the postings for any purpose.”* (57)

However, different social media sites give different levels of public access to content; for example, access to Facebook content can be restricted to predefined “friends,” whereas public Twitter posts are viewable to anyone, even people without a Twitter account. This leads researchers to question how they should approach the posted data (61).

“*The nature of new media itself blurs the boundary between public and private. Should what is posted be considered the equivalent of a personal diary or a newspaper? In some cases, the answer may be obvious but often it will not be.”* (61)

The ESOMAR guidelines suggest that:

“*Researchers should not copy or scrape content within private areas, even if they have permission of the site owner. If researchers do so, it should be made clear to all users that this is happening and they should provide individuals with a process to be excluded from such data collection.”* (38)

Adding to the complexity of blurred boundaries, public opinion should also be considered. Some social media users believed that information posted on the internet is in the public domain, thus removing the need for consent, and were also likely to think that they had forfeited the right to privacy (34).

“*I don't pay to use Twitter. I sort of signed up with the expectations that it's a free site and you just kind of throw things out publicly, [so] I don't really have an expectation that anything that I post is going to remain private.”* (Control Group, 29, male) (34)

The concept of privacy varied for individuals and was often framed by generational, cultural, and social norms (37, 58). Additionally, what is considered public and private is continuously changing, even within individual conceptions (37, 59). One issue that arose was the idea that just because something has been published, it does not mean the user expects their content to be re-used for any other purpose.

“*People may operate in public spaces but maintain strong perceptions or expectations of privacy. Or, they may acknowledge that the substance of their communication is public, but that the specific context in which it appears implies restrictions on how that information is – or ought to be – used by other parties*.” (37)

“*Data may be public (or semi-public) but this does not simplistically equate with full permission being given for all uses*.” (49)

Assuming that content posted in public implies consent for re-use also assumes that social media users have had agency over the settings on their social media account. However, there is strong evidence that users lack knowledge of social media privacy settings (46, 58, 60). This is because privacy settings can be difficult to navigate and lack transparency (46, 58). There is evidence that social media users are not always adequately equipped with digital knowledge to operate privacy settings and protect their data. This suggests that just because a post is public, the researcher cannot assume the creator of the post deliberately made it public (46).

“*For example, Facebook's privacy settings are problematic because they are opaque and dependent on the user's self-education.”* (46)

“*It was felt that Facebook often blurs the lines between what is public and private, and this lack of distinction is not made at all clear to Facebook users, e.g. the fact that ‘private' posts can be made public by re-posting*.” (41)

“*One can only properly grasp how to maximize privacy by knowing precisely what the default settings imply and what the users have signed up for … However, these privacy settings are not particularly user friendly.”* (58)

Some participants expressed a lack of understanding of how privacy settings work and believed that they should not be forced into research because of that (35, 36). In Moreno et al. (36), participants expressed confusion about whether their Facebook settings were private or public and, after discussion in the group, made comments such as:

“*Yes, so that means my Facebook is public right now? I don't want that.”* (Facebook user) (36)

“*I guess I'm surprised because I thought it was private.”* (Facebook user) (36)

“*There are quite a few people who are late to join to Facebook or are of a generation who don't know how to use it … they shouldn't be punished for that.”* (Focus Group 7) (35)

This sense of confusion may especially be to be true for users of Facebook, where users can control to some extent which other users can see their posts, and may belong to closed groups for which there is reasonable expectation of privacy. For other types of social media where public accounts are the norm, such as Twitter, there is likely to be much less expectation of privacy, especially as tweets written by celebrities are regularly reposted in mainstream media. However, even with no expectation of privacy, misconceptions were also evident among users about how social media works regarding permanence of posts, how far back data can reach, who can access the posts, and how data can be analyzed (34).

“*I would say definitely*. <*chuckles*> *Maybe it's because I'm young, so I started into social media when I was younger, like really young. So every once in a while, I'll go through [and delete].”* (Control Group, 21, female) (34)

“*I would say most of the time I'm not afraid to rock the boat. But I mean, Twitter won't let you scroll back that far, so I'm not super concerned.”* (Depression Group, 20, male) (34)

“*Are you naïve enough to think that your public tweet is going to be seen by like a million people? I mean sure, it's public. Anyone could go and find it, or search for it, or whatever. I mean, but it's not like Beyoncé tweeting is the same as me tweeting.”* (Depression Group, 54, male) (34)

However, participants in qualitative studies expressed that consent was implied and the data should not be considered private if posters had failed to protect the data through privacy settings (34–36).

“*And I don't think it's bad that you went and looked at people's profiles, ‘cause if they have them open, it's their choice.”* (Facebook user) (36)

“*I think it's the own person's fault for having a public profile because it's out there.”* (Focus Group 8) (35)

This assertion becomes especially difficult as Facebook and other platforms have been known to periodically redesign the privacy settings. Even if users are required to formally accept changes that may have affected their existing privacy settings, there is no guarantee they have comprehended, or even read updated terms and conditions. Thus, users may remain unaware of such changes. This means researchers should consider that users may not be in full control of their privacy settings and that the settings that users have chosen (or left) may not reflect their actual perception of privacy.

#### Consent—Should Users Be Asked?

Following directly from the debate on whether data could be considered public or private was the question of whether social media users should be asked for consent to re-use their posted content for the new purpose of health research. Commentators were divided between arguing for and against acquiring consent from individual users (46, 71, 75).

Many researchers followed the established notion that consent was not required to collect information already in the public domain (45, 56, 72). This position followed from the assumption that data mining is a form of secondary data analysis of publicly available material; therefore, as long as the data are freely available and log-in details are not required to view the data, consent is not required (39). Also discussed was whether social media users were “participants” or “authors of public written content” (38, 47). There were also considerations of the practicality of gathering consent, with it being impractical to gain opt-in consent from large numbers of users.

“*Individual informed consent is impractical for research involving large datasets. In these cases researchers should ensure data use is in line with terms and conditions and care should be taken to protect the identity of users*.” (39)

In all qualitative studies of social media users' views, there were participants who felt consent was not required. Some participants acknowledged that the internet is a public domain, and therefore posted data are freely available for anyone to see and use, including researchers (33–36).

“*That's a good way to do [the study]. Because if people are publicly showing their pictures, then it's, like, open for anyone to see.”* (Facebook user) (36)

“*As the information is posted on the very public Internet, I don't think there is a need for permission to use the posts.”* (Participant 10) (33)

Although this second quote indicates that some users feel the internet is public, not every user may agree, especially given the nuance of difficulty over privacy settings described above. The contrasting view was that users' consent should be solicited. Some participants in the qualitative studies felt that consent should be required (33–35), rationalizing this because the data would be used for a purpose other than the one the user originally intended (35).

“*I reckon they should ask first ‘cos they have only posted on a public site like you are posting it for people to see not for them to take the information.”* (Focus Group 5) (35)

Social media users acknowledged that consent for the re-use of their posted data was often required for acceptance of the social media website's terms and conditions (34, 35), and this was often the view taken by commentators. However, often these policies are not read by users, especially adolescents (35). Furthermore, participants felt that the terms and conditions policy was inaccessible, as the blanket language used was often difficult to understand and lacked transparency (34), and commentators agreed that signing terms and conditions did not constitute informed consent as understood in traditional research methodologies (43).

“*When consent is sought through a terms and conditions document upon entry to a particular social media site, young people may be unlikely to read it; “I don't think anybody reads them.”* (Focus Group 1) (35)

*Respondents did not feel as though simple blanket language in the “terms and conditions” constituted transparency. Such language was confusing and buried in what one participant terms, “a wall of text that no one ever reads.”* (Twitter user) (34)

“*Whilst there is a fair and lawful process for analysing social media data on quantitative scale, this is not synonymous with user consent to be included in a research project.”* (43)

Where data are not freely available without a log-in (e.g., closed Facebook groups), then there was agreement that consent should be sought before their data are used, as there is an expectation of privacy in these groups. However, a policy of gaining consent in these cases may be considered unworkable, given the logistical difficulties encountered with so many potential participants (66, 73). A suggestion attempting to mitigate the need for individual consent is that a “group consent” can be acquired, with or without an opt-out for members of the group (66); however, this too has its critics:

“*Even when conducting research on a large community that possesses a distinctive identity, such as the black community or the cancer-survivor community, obtaining the consent of the group as a whole is futile. Who can truly speak on behalf of the group of cancer survivors? This is an unanswerable question.”* (62)

There was some consensus that consent should be gained for use of data posted by vulnerable groups, including children. Children are one group that often require special forms of protection. This is often sought by gaining the consent of parents or guardians, as children do not always have adequate decision-making capacities. This poses a greater level of difficulty in the realm of online research, as adolescents can feel insulted at the notion of having to ask their parents for permission (69). Children may also be less likely to understand the full implications of posting content publicly and its possible reach (62). Also, it is not always possible to identify that users may be underage or vulnerable, so again, it is not possible to have a blanket policy for this. Rather, what constitutes the best form of consent will be best decided for the individual study.

One further issue is that of deleted posts. Users can subsequently delete their posts from the social media platform, and this could happen after the post has already been captured in the research. This might imply that consent for use of posts has been withdrawn. Researchers should plan in advance how to manage this issue. One plan might be to check that all quotes of individual posts still exist prior to publication of the results. If the quoted post has been deleted, it should be removed from the report (39).

#### Anonymity—Users Should Not Be Identifiable

There was a consensus throughout reports and articles that researchers have an important duty to maintain the anonymity and protect the identity of posters of social media content (65), throughout the analysis and especially in any publication of results. Participants in qualitative studies also highlighted the importance of ensuring the anonymity of social media users when posts were re-used for research purposes (33–36). Some participants felt that as long as the anonymity of the poster was assured, consent to use the data was not needed (33).

“*If you're using the data in some kind of statistical analysis – and not quoting directly the posting then I'd say no permission is probably needed.”* (Participant 10) (33)

“*As long as it's de-identified, that's all I really care about.”* (Focus Group 1) (35)

Throughout the literature, commentators have suggested various ways to achieve anonymity. For example, the data should be locally encrypted (58), identifiable information should be removed prior to publication (61), users' identity should be hidden through disguise (e.g., using pseudonyms or synthetic quotes) (59), and data aggregation methods should be applied (53). Participants in qualitative studies offered suggestions of acceptable ways to ensure their anonymity was maintained. These included aggregation of data (34), making generalizations, and removing identifiable information (33).

“*I'm OK as long as we can, you know, figure out ways to keep the data anonymous and completely, highly aggregated.”* (Depression Group, 47, male) (34)

“*Few people would have a problem with generalised and anonymised references.”* (Participant 17) (33)

“*[Permission isn't needed] as long as you don't identify the poster by more than sex, age, and type of diabetic.”* (Participant 20) (33)

In the social media context, there may be a greater risk to an individual's confidentiality and anonymity compared to conventional research because search engines may be able to detect the original post of a user when a key phase or quote is published in a research article (37, 41, 74). This could allow for identification of the user's personal profile, which opens the possibility for them to be contacted via personal messages through the website (51, 56, 61, 63, 68, 69).

To avoid this risk, commentators have suggested avoiding the use of direct quotes from a user's post (38, 51, 63) or paraphrasing quotes if it is felt necessary to include this type of data in the writeup (42, 56, 59, 61). A third possibility is to synthesize quotes to illustrate a finding, which are based on principles within the data.

“*To better maintain the principle of respect for person while presenting verifiable data, we recommend that researchers paraphrase users' comments*.” (56).

Participants in qualitative studies also identified the threat from the use of direct quotes from participants' posts (33).

“*If you want to use actual quotes from people that's a different matter as even if you make the quote anonymous in your research it will be quite easy to find the author simply by typing in key phrases into Google which will then give links back to [the forum].”* (Participant 29) (33)

The ESOMAR guidelines recommend how to deal with this problem:

“*If researchers wish to quote publicly made comments, they must first check if the user's identity can be easily discoverable using online search services. If it can, they must make reasonable efforts to either seek permission from the user to quote them or mask the comment to such an extent that the identity of the user cannot be obtained.”* (38)

There have also been concerns raised that even data aggregation is not fool-proof in terms of disguising group identities. For example, in one case study, an adversary found the identity of an anonymized university, when only aggregated data were presented. This was due to the uniqueness of the information given about the university that the participants attended. If the course and year group of students were also identified, it would then be a short step for an adversary with inside knowledge to re-identify individuals [(63), referencing (16)].

The importance of protecting anonymity was of greater concern to certain populations, particularly those who live with a stigmatized diagnosis or who are part of a vulnerable group (such as adolescents) (34, 35). Stigmatization and bullying were two key concerns:

“*Once you've got the taint of depression – mental illness at all in our society, it's an uphill battle. Even now, people in my family are like, ‘Oh, you sound cranky. Have you taken your meds?”* (Depression Group, 33, male) (34)

“*De-identification of social media posts was crucial to minimise negative ramifications; “If you do write something on there, it is going back to maybe someone you know, you could get bullied for that reason.””* (Focus Group 3) (35)

The ESOMAR report also stated a list of features of social media content where it may be especially important to protect user identities: if the topic being discussed is sensitive or personal; if abusive language is used; if it includes anything against the law; if it includes anything embarrassing or is likely to impact career opportunities; and if it includes any personally identifiable information or data about others that is not already public (38).

#### Weighing Harms Against Benefits

“*Researchers have an obligation to avoid causing physical, emotional or psychological harm to participants.”* (40)

According to Beauchamp and Childress' principles of research ethics, researchers have an obligation to ensure no harm comes to participants, and that the research will have potential benefits for the target group (21). The risk of harm is greatest when “*a social media user's privacy and anonymity have been breached*” (42). After considering and minimizing the risks of a breach in confidentiality, researchers must also identify any further sources of harm that their study could precipitate (37, 38, 41, 42, 46). This is often difficult for researchers mining social media data, as they need to predict sources of harm ahead of publication, which are often not apparent (74).

“*Is the project a potential source of harm? … it may be difficult to identify “harm.” Researchers have to be thoughtful about any potential harm that their research might incur by being sensitive to the content extracted from social media websites, the degree and context of content exposure, and the authenticity of the material used.”* (46)

“*Does the connection between one's online data and his or her physical person enable psychological, economic, or physical harm?”* (37)

Throughout the literature, commentators identified several sources of harm that should be considered by online researchers. Possible harms include the following: blurring of personal and professional boundaries; creating a culture of mistrust between researchers and users (which can form when taking data without consent) (54); leaving the user at risk of “abuse” (40); embarrassment, reputational damage, or prosecution (42) and abusing the power imbalance that can exist between researcher and user (56).

“*Power, especially the power differential between the researcher and the researched, must be considered. Where the power imbalance is abused, there is a significant threat to justice and the potential for harm.”* (56)

“*Participants felt that being identifiable in research could lead to unsolicited attention online and, more seriously, ‘abuse'. This might be from people they knew, or from organisations that could ‘exploit' them.”* (40)

Special care to assess risks and benefits must be taken in certain situations, including vulnerable groups such as children, and with sensitive issues that could lead to stigmatization (52, 60, 74). Harms could result from users' being re-identified within the research, or from the publication of sensitive findings that could harm an entire group (58). This places a responsibility on researchers to consider methods to ensure that individuals' anonymity is maintained (60), along with being sympathetic to the generalizability of their findings (52).

“*Vigilance is required when conducting social media research on sensitive topics that might permit the identification of participants, resulting in stigmatization; the dissemination of findings that could harm an individual or social group; challenges to an individual's values or beliefs; and instances of bullying and abuse. Such research risks inducing or exacerbating emotional distress.”* (58)

When re-using potentially personally identifiable data, the benefits of the research must be justified (46, 51), especially considering the introduction of the General Data Protection Regulation (GDPR), as this legislation provides a legal responsibility for European researchers to ensure they provide justification of the benefits their research will provide (58). Researchers should consider the risk–benefit relationship within each study, so that information discovery that can contribute to patient care and well-being can proceed, while causing minimal harm to users (56). One way of ensuring a thorough consideration of risks and harms, which may not be obvious to researchers without lived experience of a condition, is to include social media users from the target population in the study team or consult with them before the research is conducted.

#### Research for Public Benefit

An overarching theme that emerged from the qualitative studies with participants was an altruistic view that if the research was being done for the greater good, and not for commercial gain, then many people were in support (33–36). Some participants were even willing to put privacy concerns aside for the greater good of the research (34).

“*Well, I mean Facebook is pretty much open to anyone, so as long as it's not for a bad intention I think it's fine.”* (Facebook user) (36)

“*When people post on the Internet, it is there for all to see. They should not complain if it can be harvested and used for the general good.”* (Participant 12) (33)

“*I can't be in a position to know all the possible things that someone could come up with, all the beneficial things, all the harmful things. I think [it represents one-percent of the issues], the whole array of things that are possible shouldn't be stopped because we're so overly worried about [privacy].”* (Depression Group, 54, male) (34)

“*‘It could well be of benefit to the, you know, the people who deal with these kind of things that, good information about domestic violence. And if there's no risk to the individuals [whose information is being used] then it's probably a good thing (Male, age 61*+*, High User)* (40).

This was felt strongly by some adolescent participants, who also felt that the research had to be done by reliable organizations, so that their words were not taken out of context (35).

“*Like having a trustworthy organisation that we know you're not going to like spin our words and make us look like bratty teenagers who just post because we can. Like try and understand it from our point of view I guess.”* (Focus Group 1) (35)

This contrasted with research being done “for profit” by private companies or to drive an agenda.

“*Research being conducted by a not-for-profit organisation, rather than for ‘commercial' reasons, was preferred for two reasons. Participants who preferred not-for-profit researchers to commercial organisations did so because the former were felt to be more ‘productive', more ‘ethical' and ‘not exploitative'. The second reason not-for-profit researchers were preferred is because participants did not like to think of their social media posts being used to generate a profit for others.”* (40)

#### Ethical Approval for Studies—Is It Needed?

Throughout the papers, there was mixed opinion regarding whether research ethics committee (REC) approval was required for studies that mined publicly available data from social media sites. A systematic review of mental health social media research papers identified that only 9 out of a total 48 papers gained REC approval, and a further 2 used a dataset that had ethical approvals in place (78); no obvious methodological differences were reported between these studies and those that did not seek ethical approvals.

A key consideration when determining if social media mining required REC approval focused on whether the social media users who posted the content are considered human research subjects and therefore participants in the research; or if their posted content can be treated as stand-alone public data of which they are authors, and therefore the research can be considered a secondary data analysis (37, 47).

“*In internet research, ‘human subject' has never been a good fit for describing many internet-based research environments*. (37)

Some researchers feel that the process of mining social media data is synonymous to observing people in a public place, such as a park, but they also identify that this perspective may not be shared by everyone.

“*The researcher believes that … individuals are aware that they are in the public sphere and potentially being observed. She seeks research ethics consultation because she recognizes that others may feel that viewing of publicly available Facebook pages is qualitatively different from observing unknown people in a park, for example.”* (73)

On the other hand, some researchers have attempted to define more clearly the meaning of human subjects research by applying a legal definition to the term “human subject.” With this definition excluding the authors of social media content, it is then suggested that this form of passive data use is exempt from the REC approval process, especially if the researcher is not interacting with users or publishing identifiable information (55, 59, 63, 76).

“*If the following conditions are met: access to the [social media websites] is public; information is identifiable, but not private; and information gathering requires no interaction with the person who posted it online; then presumably the proposed project does not constitute human subjects research.”* (63)

“*Since most social media projects rely on publicly available data, and do not include interventions or interactions with the population, they may qualify for IRB [internal review board] exempt status”* (47)

Where researchers do seek ethical approvals from a committee, they should not assume that this absolves them of considering all the ethical issues around the project themselves (such as user anonymity, and risk/benefit ratios):

“*Many ethics boards do not understand the processes of mining and anonymizing Big Data, let alone the errors that can cause data to become personally identifiable. Accountability requires rigorous thinking about the ramifications of Big Data, rather than assuming that ethics boards will necessarily do the work of ensuring that people are protected*.” (49)

#### Legal Issues and Terms and Conditions of Sites

Fewer articles mentioned this theme, and it was found most commonly in articles and reports found through the gray literature search. While automated technological tools “*can collect, clean, store and analyse large volumes of data at high velocity*” (39), researchers are not always permitted to scrape data in this way by social media sites' rules (50). Researchers should be clear on the constraints within social media sites' terms and conditions and should make sure they are operating within the law.

“*It is important for researchers to take the time to read user agreements for social media platforms as they govern what practices are permissible and provide guidance on publishing posts.”* (44)

Several guidelines advise researchers to abide by the regulations of the website that they are mining data from (37, 40, 42, 72), and the law (38), and to identify what users consented to at the time of data capture (41). For example, if the research is being conducted on Facebook, it explicitly states in the terms and conditions that researchers should both inform and gain the consent of the people from whom they collect data (58, 63).

“*Facebook's Statement of Rights and Responsibilities now states that, when collecting users' personal data, one must obtain consent, make it clear who is collecting the information, and declare how the data will be used.”* (58)

By agreeing to Twitter's terms and service agreement, users consent for their information to be collected and used by third parties (44). Researchers using Twitter data often justify their collection and analysis of Twitter posts because users have signed this agreement. However, the Twitter terms and conditions do not allow scraping or downloading of tweets (instead, researchers should use an approved Twitter API), and therefore, researchers who scrape data may be in contravention of Twitter's terms and conditions (44). A further consideration is that Twitter users retain the “right to be forgotten” (77), and this right complicates the publication of direct quotes, especially without consent, as these cannot easily be removed from peer-reviewed publications. Other sites such as Reddit also have official APIs for accessing posts and associated metadata for use in research.

### Governance of Data, Annotations, Algorithms, and Linkage

An emerging theme, populated only by the most recent articles and those with an NLP focus, suggested that researchers should focus on transparency of methods, and good governance practices with regard to datasets, annotations, and linkage.

There is a tension over the datasets created following the harvesting of social media data. Two arguments have been made, both in favor of sharing openly datasets and annotations, and advocating that datasets should be protected in case they contain potentially sensitive data. Datasets where there is a risk of user identity disclosure may be placed on a protected server for example (47).

“*We strongly encourage researchers to share datasets and annotations they have created so that others can replicate research findings and develop new uses for existing datasets… However, where there may be risk to users, data should not be shared blindly without concern for how it will be used.”* (47)

While sharing annotated datasets substantially reduces the burden on other researchers to create and annotate new sets, annotations of the data should also be considered as potentially sensitive:

“*Domain experts may manually label users for different medical conditions based on their public statements. These annotations, either manually identified or automatically extracted, may be considered sensitive user information even when derived from public data.”* (47)

It is recommended that annotations be stored separately from the raw data where possible.

To reduce the likelihood of re-identification of or harm to users, it is recommended to remove the author's name and @tag from the dataset, strip out other named persons or place names, remove metadata such as geolocation, generalize data such as locations to large group categories (e.g., city rather than street name), and identify where “*the need for creating derived characteristics is crucial to a project, and not running these algorithms as standard.”* (43)

A further level of transparency is needed around the data processing and information extraction methods used in the research. While data science methods can be couched in claims of objectivity, researchers should be aware that biases may be introduced by their algorithms or case identification, entity recognition, or relationship extraction methods. It is also important that these methods are made available to the public and social media users in a culture of transparency:

“*Machine learning algorithms do not learn perfectly predictive models. Errors and misclassifications will be made, and this should be accounted for by the researcher.”* (47)

“*If using software that enhances Twitter data, ensure algorithms that derive characteristics and classify users are open to researchers and research subjects. The accuracy, configurations and threshold settings of algorithms should be made public to ensure reproducible research.”* (77)

“*Many are not aware of the multiplicity of agents and algorithms currently gathering and storing their data for future use. Researchers are rarely in a user's imagined audience.”* (49)

Special caution should be used when using “off-the-shelf” algorithms, which are not well-understood by the researcher:

“*Data scraping without context may result in potentially inaccurate algorithms that may get reported and reused in application, leading to potentially harmful consequences.”* (50)

Researchers should be very cautious when linking data across sites or to other data sources. While users may share data publicly on multiple platforms, they may not intend for combinations of data across platforms to be public (61). Caution should especially be used if trying to identify the same user posting on separate sites or platforms, as they may not wish to be identifiable on all platforms (e.g., on Twitter vs. an anonymous patient forum). Given the high likelihood of making individuals more identifiable by linking data across different sources, REC approvals should be sought for this activity.

## Discussion

Our review demonstrates key ethical issues in approaching text mining of social media data for health research and is relevant to all NLP and text-mining researchers who engage in this endeavor. Like previous reviews and guidelines, we have shown the existence of a complex intertwined matrix of ethical considerations around the use of social media data for research purposes. We have extended previous work by showing some themes that are specific to analysts and computer scientists who employ algorithms and other methods for processing multiple documents automatically.

The key issue for academic researchers that may determine whether consent or ethical review is needed is to reach consensus on whether social media users are considered human subjects within the research, or whether their data are assumed to be public data of which the researcher is undertaking a secondary analysis. Many commentators' view was that much social media is in the public domain, the public or private nature of which is controlled by the user via their account privacy settings. Therefore, if they are freely displaying the data in an observable public domain, they are relinquishing the privacy rights of that data, and the data can be considered public. However, this should be balanced by researcher consideration of how easy it is for users to control their privacy settings in the platform from which the data are being collected.

Informed consent is a voluntary agreement by a participant with mental capacity who understands the full consequences of participating in research. It is one of the most critical aspects of research ethics (79), which likely accounts for the strong theme discussing issues around consent in this review. The principles of informed consent are embedded in the most influential research ethics reports, such as The Nuremberg Code, The Declaration of Helsinki, and The Belmont Report (20). In the case of social media data being considered public, experts generally agreed that consent was not needed, except in special cases involving vulnerable populations and sensitive issues.

However, social media users were more divided on the requirement for consent, as they acknowledged that privacy settings were challenging to navigate. Often social media platforms update their terms of use or privacy controls, meaning users must go back and navigate a new system to ensure their privacy choices are still set correctly. These shifting sands may be considered—as in Gibson's theory of affordances (80), which is used in science and technology studies and other related fields (81, 82)—as agency on the part of the social media platform. In this example, changes to the technical properties of platforms may function to constrain agency by the user, ensuring that more public content remains available by default, and connections between users can be maximized (83).

Another issue raised in the arguments against gaining consent was that it is often impractical to do so when such large datasets are being used. Some previous commentators have discussed whether impracticality in gaining consent can be considered a justification for not doing so. This has been discussed in relation to clinical trials of emergency treatment, for example, but also in circumstances where requiring consent will reduce inclusion to the study to the most engaged or empowered sectors of society, such as is likely with big datasets in health (84). This then affects data quality and results in bias in participant selection and possibly the most disenfranchised sectors of society remaining under-researched. In addition, it has been noted that users with health conditions actively manage how their identities are presented online, with privacy in mind (85). For example, those who suspect their data may be used to infer their health status, for example, for targeted advertising, may deliberately deploy strategies to mask their underlying condition (e.g., by filtering or changing the content or frequency of posts) (86). This again will have an impact on data quality and reliability.

Despite data often being considered public, experts and users expressed the importance of ensuring the anonymity of social media users in project publications, and acknowledged that this can be difficult to achieve if full direct quotes are printed. Therefore, researchers should take appropriate precautions to ensure anonymity by employing methods such as removing identifiable information, disguising identities, aggregating data, making generalizations, and creating synthetic quotes. Anonymization of the data also contributes to the argument that consent is not needed: within the 2001 bulletin of the Declaration of Helsinki, it states “*Medical research involving human subjects includes research on identifiable human material or identifiable data”* (87), thus removing, or not collecting, users' identifiers contributes to the determination that social media mining research does not fit the criteria of human subject research.

Even if under certain conditions, social media mining does not fit the mold of human subjects research, this does not mean it is free of ethical issues. The issues involved may not be addressed by traditional ethical frameworks (88) and new, creative ways of thinking about research ethics may be needed. One potential method is The Ethics of Care Theory (89). This is a theory that has been developed from feminist thought toward the morals of caring for others. Unlike more traditional basis of ethics, it focuses on the relationships in the research, acknowledging the differences in power that exist along with vulnerability. It also emphasizes the ethical decisions that need to take place in project design (90).

In line with this theory, our findings suggest that researchers should consider and identify potential harms that could arise from the use of social media data, whether these relate to individuals whose data was used, or to a patient group as a whole (such as those posting about stigmatized issues), or to the relationships and trust between social media users and the research community. Within our findings, there was reference to special consideration that should be awarded to vulnerable groups and stigmatized issues, but it can be difficult to define what these terms encompass. More thought should be given to defining these groups, but it is likely that researchers will need to be reflective and determine this on a project-by-project basis. Including social media users with lived experience of the conditions under study within research teams will help researchers to be more reflective about the potential for stigmatization or harm, which could result from their work. Harms should ideally be explored prior to research commencing, by consulting with all relevant stakeholders. To ensure widespread approbation of the research, researchers should make it clear when the primary goal is public benefit, rather than for private profit or to further an agenda, and the routes to benefits should be made transparent in the dissemination of the research.

Research quality and transparency are additional issues that contribute to the overall ethical nature of scientific studies. It is especially important that text-mining researchers ensure the quality of their work by developing or using high-quality techniques and examining carefully any limitations or biases in their understanding of the data and the context of its production, or in the transparency and quality of their data scraping and analysis methods. A lack of care and rigor in these elements of a study would make their work scientifically concerning even without other ethical issues (50).

### Strengths and Limitations

We identified an extensive collection of peer-reviewed articles, commentaries, editorials, and gray literature reports, but may not have captured every piece of writing on this issue. The search specifications for the three searches were challenging, due to the interdisciplinary nature of this review (computer science, medical research, and ethics), meaning that MeSH terms could not be used. Therefore, it is possible that some terms may have been neglected, for example, while we used the terms “discussion forum” in relation to internet forums, we did not specify sites such as Reddit, Linked-in, Instagram, WeChat, or YouTube; thus, it is possible that we missed papers focusing on specific sites. This may have limited the scope of themes, such as the legal, terms and conditions theme, to identify issues relating to each site. Furthermore, because user demographics vary across sites, this choice may have affected the range of participants (and views) included in the review.

In addition, it is likely that this research missed some viewpoints by excluding papers not focused on health research and academic research, meaning that the guidelines formed here may be missing considerations specific to other scientific sectors such as social and market research. As our searches were limited to medical-, social science-, and life science-focused databases, we may have missed some of the computer science literature, such as papers published as conference proceedings. Some of these were picked up in the gray literature search, but there remains the possibility that some may have been missed.

Once the final articles were selected, it was not possible to assess the quality of expert commentary articles and gray literature guidelines, and public views reported were taken from a small pool of four papers, due to the lack of peer-reviewed research in this area. This likely resulted in a selection bias in the results, as the articles focused on specific populations (adolescents, mental health, and diabetes), and so the views demonstrated here may not be generalizable to all other social media users or conditions that could be studied.

While every effort was made to be neutral and data-led in this thematic analysis, due to the qualitative nature of this study, it is important to recognize the possibility of unintentional bias or subjectivity in the results because of the researchers' academic interests and knowledge. However, our results are comparable with other reviews in this field, such as Golder et al. (23).

### Recommendations, Future Work, and Conclusions

Social media research using text analytics and NLP is evolving quickly in a largely unregulated landscape (24), with many researchers acknowledging the absence and subsequent need for guidance (23, 51, 66, 68, 69, 72, 75). While, in a range of circumstances, social media text mining can legally and reasonably proceed without specific ethics committee approvals, there are certain circumstances where scrutiny from ethical committees should be sought.

Ethical approval was considered necessary for research using data from closed groups, engaging in direct contact with users, when conducting any kind of intervention through social media, if research was specifically about users who are under 18 or lack capacity, if users could be identified from the study publication or dataset, if multiple sources of data are being linked, or if, following consultation, it is assessed that there are reasonable risks of potential harms or stigmatization occurring. Likewise, researchers should gain consent from social media users in the circumstances above.

Regardless of whether formal approvals are sought, we make some additional recommendations to improve ethical standards in all text-mining research using social media data for health research purposes, including increasing public awareness about research uses of social media data; aiming for transparency in data access and analysis methods; transparency in routes to benefits for users from the research; consultation with social media users and target groups to identify and mitigate against potential harms that could arise from the research; and ensuring the anonymity of social media users by masking or synthesizing direct quotes and aggregating quantitative data. Researchers should always act within the law and abide by the social media site's terms and conditions, for example, using approved APIs to access data, such as exist for Twitter and Reddit, among other sites. The research community as a whole should foster a culture of continuous improvement in terms of technology and transparency of methods for the processing of social media data for health research (91).

Future work in this area will aim to distill out a list of recommendations or guidance for text-mining researchers that can be widely disseminated, working with national regulators and advisors. In addition, we propose consulting with social media providers to work with them to improve transparency of terms and conditions, and accessibility of guidance, for accessing and using their users' data for health research. This would help to ensure that transparent and ethical practice becomes embedded in the culture of text-mining social media data and that ethical guidance is available to all. We also note that the number of studies asking social media users for their perspective on their data being used for health research is very limited, and we would recommend more studies be conducted in this area.

## Data Availability Statement

The original contributions presented in the study are included in the article/supplementary material, further inquiries can be directed to the corresponding author/s.

## Author Contributions

EF conceived the study and carried out some searches, screening, and analysis and redrafted the paper. SS conducted searches, screening, and analysis and wrote the first draft of the paper. LH carried out some screening and commented on paper drafts. KJ commented on paper drafts. All authors agreed to be accountable for the content of the work.

## Conflict of Interest

The authors declare that the research was conducted in the absence of any commercial or financial relationships that could be construed as a potential conflict of interest.
